# Time-Course Transcriptional and Chromatin Accessibility Profiling Reveals Genes Associated With Asymmetrical Gonadal Development in Chicken Embryos

**DOI:** 10.3389/fcell.2022.832132

**Published:** 2022-03-08

**Authors:** Jianbo Li, Congjiao Sun, Jiangxia Zheng, Junying Li, Guoqiang Yi, Ning Yang

**Affiliations:** ^1^ National Engineering Laboratory for Animal Breeding and Key Laboratory of Animal Genetics, Breeding and Reproduction, Ministry of Agriculture and Rural Affairs, College of Animal Science and Technology, China Agricultural University, Beijing, China; ^2^ Shenzhen Branch, Guangdong Laboratory of Lingnan Modern Agriculture, Genome Analysis Laboratory of the Ministry of Agriculture and Rural Affairs, Agricultural Genomics Institute at Shenzhen, Chinese Academy of Agricultural Sciences, Shenzhen, China

**Keywords:** chicken, gonad, asymmetry, RNA-seq, ATAC-seq, LHX9

## Abstract

In birds, male gonads form on both sides whereas most females develop asymmetric gonads. Multiple early lines of evidence suggested that the right gonad fails to develop into a functional ovary, mainly due to differential expression of PITX2 in the gonadal epithelium. Despite some advances in recent years, the molecular mechanisms underlying asymmetric gonadal development remain unclear. Here, using bulk analysis of whole gonads, we established a relatively detailed profile of four representative stages of chicken gonadal development at the transcriptional and chromatin levels. We revealed that many candidate genes were significantly enriched in morphogenesis, meiosis and subcellular structure formation, which may be responsible for asymmetric gonadal development. Further chromatin accessibility analysis suggested that the transcriptional activities of the candidate genes might be regulated by nearby open chromatin regions, which may act as transcription factor (TF) binding sites and potential cis-regulatory elements. We found that LHX9 was a promising TF that bound to the left-biased peaks of many cell cycle-related genes. In summary, this study provides distinctive insights into the potential molecular basis underlying the asymmetric development of chicken gonads.

## Introduction

The ovary, a significant organ in humans and animals, affects sexual maturity and reproductive performance. In the poultry industry, well-developed ovaries are of great significance for improving production performance and economic benefits. In addition, gonadogenesis is also a great biomedical model for studying infertility and sex differentiation disorders in humans (Arboleda et al., 2014; Guercio and Rey, 2014). Unlike mammals, most female birds develop asymmetric gonads, that is, only the left gonad develops into a functional ovary, whereas the right gonad remains rudimentary (Smith and Sinclair, 2004; González-Morán, 2011). Although several investigations have focused on this particularly intriguing phenomenon, the distinct developmental patterns that occur on the two sides are still elusive (Scheider et al., 2014; Wan et al., 2017; Yu et al., 2017; Zou et al., 2020). Thus, there is an urgent need to elucidate the molecular mechanisms underlying asymmetric gonadal development in female embryos.

In chicken embryos, gonadogenesis begins at approximately 72 h of development (Hamburger Hamilton Stage 23, HH23) (Hamburger and Hamilton, 1951). As development progresses, the morphology of the bilateral gonads in females is initially similar at embryonic days 5.5 (E5.5, HH28), but by E6.5 (HH30), the gonads are distinctly different (Guioli et al., 2014). It is widely accepted that the gonads contain two layers, namely, the cortex and medulla (Smith and Sinclair, 2004). The left gonad develops a side-dependent thicker cortex and ultimately differentiates into a functional ovary, while the right ovary is surrounded by a thin, flat epithelial layer and later undergoes developmental arrest (Smith et al., 2007; González-Morán, 2011; Intarapat and Stern, 2013). Almost all primordial germ cells (PGCs) are located in the cortex, and a few are scattered in the medulla. Indeed, due to the preferential migration, the number of PGCs present in the gonads exhibits an obvious sex-independent asymmetric distribution, and more PGCs are found on the left side at approximately 50 h (HH15) to E16 (HH42) and before hatching (Van Limborgh, 1968; Nakamura et al., 2007; Intarapat and Stern, 2013; de Melo Bernardo et al., 2015). Additionally, the PGCs in the cortex exhibit faster proliferation rates at E5-E6 (HH27-HH29) (Ishimaru et al., 2008). However, the PGCs in the right gonad fail to enter meiosis and disappear posthatching. This fate is similar to that of germ cells in the medulla of the left ovary (Guioli et al., 2014). Therefore, increased proliferation in the left cortex is primarily responsible for asymmetric ovarian development. Although this anatomical developmental process is relatively clear, the underlying functional drivers and regulatory network remain to be further studied.

The side-dependent morphological development of the embryonic gonad is regulated by multiple side-biased genes, which were described in a previous study (Monsoro-Burq and Levin, 2018). For instance, PITX2, which is strongly expressed in the gonadal epithelium, controls gonadal morphogenesis and rescues right gonad fate by affecting the RA pathway to regulate target gene expression and stimulate cell proliferation (Guioli and Lovell-Badge, 2007; Ishimaru et al., 2008; Rodríguez-León et al., 2008). In addition, several genes, such as FET-1, BMP7, R-Spondin1 and OVEX1, have been reported to be expressed in the developing gonads of chickens in a side-dependent manner (Reed and Sinclair, 2002; Hoshino et al., 2005; Smith et al., 2008b; Carré-Eusèbe et al., 2009). Despite the identification of putative candidate genes, the mechanisms by which these genes regulate asymmetric gonadal development are not clear. Accessible regions of the genome are considered primary regulatory elements, such as promoters and enhancers, and TFs binding sites to modulate gene expression and further determine cell fate (Buenrostro et al., 2013; Rivera and Ren, 2013; Burton and Torres-Padilla, 2014; Wu et al., 2016). Accordingly, it is essential to elucidate the dynamic patterns and regulatory functions of open chromatin regions across asymmetric gonadal development.

To obtain a better understanding of this progress, we explored the transcriptional and epigenetic changes that occur during asymmetric gonadal development by high-throughput paired-end RNA sequencing (RNA-seq) and Assay for Transposase Accessible Chromatin with high-throughput sequencing (ATAC-seq). The present work generated a high-resolution developmental profile of chicken gonads at the gene expression and chromatin accessibility levels, identified numerous candidate genes and cis-regulatory elements, and showed functional relationships between these elements. It should be noted that we proposed that a promising TF, LHX9, was enriched in left side-specific open chromatin regions and might be recruited by certain pioneer TFs to activate left ovary development; this hypothesis was initially supported by *in vitro* experiments. In summary, our findings provide important insight into the earlier steps of asymmetric gonadal development in female chicken embryos and will be beneficial for future studies on human infertility.

## Materials and Methods

### Ethics Statement

The experiments were approved by the Animal Welfare Committee of China Agricultural University and performed in accordance with the protocol outlined in the “Guide for Care and Use of Laboratory Animals” (China Agricultural University).

### Embryo Tissue Collection

Fertilized eggs were obtained from a pure line of *White Leghorns* raised in the Experimental Base of Poultry Genetic Resources and Breeding, College of Animal Science and Technology, China Agricultural University. The eggs were incubated in an automated egg incubator at 37.8°C and 60% relative humidity, and the eggs were rotated every 2 h. Once the eggs reached E4.5 (HH25), E5.5 (HH28), E7 (HH31) and E10 (HH36), respectively, the left and right gonads of the chicken embryo were separately harvested. The remaining embryo tissues were collected to determine sex by a direct PCR kit (TransGen Biotech, Beijing, China) using *CHD1* primers (Supplementary Table S1). Additionally, three biological replicate samples were collected from each side and each stage.

### RNA Isolation, Library Preparation and Sequencing

The left and right gonads were dissected. One embryonic gonad was used for each biological replicate. The gonads were immediately placed in RNAlater™ Stabilization Solution (Thermo Fisher Scientific, Waltham, MA, United States) and stored at −20°C. Total RNA was extracted with TRIzol reagent (Thermo Fisher Scientific, Waltham, MA, United States) following the manufacturer’s instructions. The RNA concentration and purity were measured using a NanoDrop 2000 spectrophotometer (Thermo Fisher Scientific, Waltham, MA, United States). The integrity of the RNA was determined using an Agilent 2100 Bioanalyzer (Agilent Technologies, CA, United States). To construct the libraries, reverse transcription and amplification of the transcripts were performed following the Smart-seq2 protocol. The libraries were sequenced on the MGI-500 platform (150 bp paired-end, PE150).

### ATAC Library Preparation and Sequencing

Each biological replicate for four developmental stages comprised one embryonic gonad. The samples were dissected as described above and then preserved in a cryopreservation tube with DMEM/F12 (GIBCO, Grand Island, NY, United States) supplemented with 10% fetal bovine serum (GIBCO, Grand Island, NY, United States) and 10% DMSO (Sigma-Aldrich). The cryopreservation tubes containing the samples were immediately placed in a cryopreservation box at 4°C for 1 h and then -80°C overnight. The nuclei were isolated from the frozen tissues according to previously described methods (Corces et al., 2017). The nuclei were pelleted at 500 RCF for 10 min at 4°C in a fixed angle centrifuge. The cell pellets were resuspended in 50 μL of transposition mixture containing 25 μL 2x TD buffer, 2.5 μL transposase (100 nM final), 16.5 μL phosphate-buffered saline (PBS; GIBCO, Grand Island, NY, United States), 0.5 μL 1% digitonin, 0.5 μL 10% Tween-20 and 5 μL H2O and incubated at 37°C for 30 min. A cleanup reaction was performed to obtain the DNA for the PCR amplification of transposed fragments with 29 cycles. The PCR products were quantified with the KAPA Library Quantification Kit (#KK4844; Kapa Biosystems) and then sequenced using PE150 reads on the Illumina Novaseq 6,000 platform.

### RNA-Seq Data Analysis

Paired-end RNA-seq reads were aligned to the chicken reference genome (GRCg6a) using HISAT2 (Kim et al., 2015) with default parameters before using HTSeq-count (Anders et al., 2015) to count the reads mapped to individual genes. The generated count matrix was used as input for the DESeq2 (Love et al., 2014) package to identify DEGs between left and right gonads. Candidates with greater than 1.5-fold changes at adjusted p-values  <  0.1 were considered significant DEGs.

### ATAC-Seq Data Processing

The PE150 ATAC-seq reads were trimmed into PE50 using Perl scripts, and then, the PE50 ATAC-seq reads were mapped to the chicken reference genome (GRCg6a) assembly using Burrows–Wheeler Aligner (BWA) (Li and Durbin, 2009) with default parameters. SAM files were converted to the BAM format using Samtools (Li et al., 2009), and PCR duplicates were removed using the Picard MarkDuplicates option to generate filtered BAM files. The peaks were called using MACS2 (Zhang et al., 2008) in each sample and using the filtered BAM files with the parameters (-g 9.6e8 -q 0.01 -B --SPMR --nomodel -shift -100 -extsize 200). Referring to the proportion of the effective genome sizes of humans and mice, we adjusted the parameters of –g in chickens. All the alignment files were extended to 200 bp and scaled to RPKM-normalized read coverage files using deepTools (Ramírez et al., 2014) for visualization. The library size factors estimated by the DESeq2 package were applied to the RPKM values to compare binding profiles between different samples in an unbiased manner. DARs were detected using DESeq2 with a fold change less than 1.5 and q-value below 0.1. The HOMER tool (http://homer.salk.edu/homer/motif/) was used to detect the motifs. The BEDtools suite (https://bedtools.readthedocs.io/en/latest/content/bedtools-suite.html) was used to test overlap and enrichment between different intervals.

### Functional Annotation

Using biomaRt, we identified homologs of chicken DEGs in humans. Functional analysis of these homologs was performed using the Metascape online tool (http://metascape.org). The Gene Ontology (GO) terms for biological process, cellular component, and molecular function categories, as well as Kyoto Encyclopedia of Genes and Genomes (KEGG) pathways, were enriched based on the Metascape online tool with default parameters.

### Cell Separation and Culture

The left gonads were removed and placed in cold PBS when the fertile chicken eggs reached E7 (HH31). Samples were dispersed by incubation with trypsin 0.25% (GIBCO, Grand Island, NY, United States) at 37°C for 10 min with constant shaking. The digestions were stopped by the addition of culture medium containing DMEM/F12 with 10% (v/v) fetal bovine serum and 1% penicillin/streptomycin (GIBCO, Grand Island, NY, United States), and the samples were passed through a cell strainer (40 μm) and collected into 50-ml tubes. Subsequently, the cells were washed with PBS and seeded in cell culture plates. Sex identification was carried out by PCR as described above. Cells, derived from 15 gonads, were pooled for each biological replicate.

### Plasmid Construction, Transfection and qRT-PCR

Gonadal cDNA was synthesized using the First-Strand Synthesis kit (Takara, Japan) and PrimeScript™ RT reagent kit with gDNA Eraser (Takara, Japan). The coding sequence of *LHX9* was amplified with PCR primers (Supplementary Table S2) using Polymerase PrimeSTAR (Takara, Japan). The amplified fragments were digested with the restriction enzymes MluI and HindIII (NEB) and cloned into the pcDNA3.1 vector using T4 DNA ligase (NEB). When the cells reached 40% confluence, they were transfected with the plasmids by using Fugene HD (Promega, Madison, WI) according to the manufacturer’s instructions. After transfection for 48 h, the cells were collected for RNA extraction, and cDNA was synthesized as described above. qRT-PCR was performed with an ABI 7500 system (Applied Biosystems) using the TB Green^®^ Premix Ex Taq™ Kit (Takara, Japan) according the manufacturer’s instructions. *β-actin* was used as the internal control, and the sequences of the gene-specific primers are listed in Supplementary Table S3.

### Statistics Analysis

Statistical analyses were performed using the SPSS software (version 25.0; IBM, Chicago, IL, United States). The data are expressed as the mean ± SD (standard deviation) and were analyzed using a two-tailed Student’s t-test, and at least three replicates were conducted in multiple independent experiments. The differences were considered to be statistically significant at a *p*-value < 0.05.

## Results

### Asymmetric Development of Gonads

**FIGURE 1:**
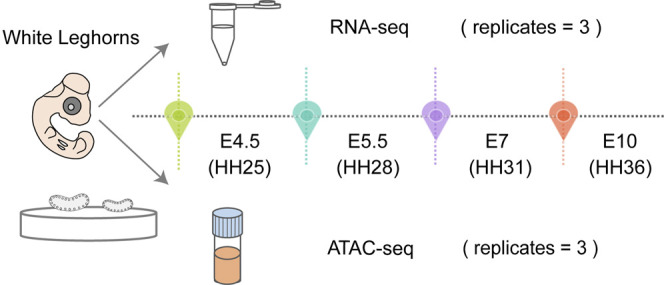
Schematic representation of chicken embryos with left and right gonads dissected for RNA-seq and ATAC-seq at each time point in triplicate.

Gonadal morphological characteristics were assessed at four representative stages during embryonic development, including E4.5 (HH25), E5.5 (HH28), E7 (HH31) and E10 (HH36). To ensure a comprehensive understanding of asymmetric gonadal development, bilateral gonads were collected at each stage in triplicate for RNA-seq and ATAC-seq (Figure 1) The bilateral gonads emerge on the ventral surface of each mesonephros and continue to increase in size at different rates during embryonic development. The gonadal morphological appearance of the female chicken embryos was initially similar on the left and right sides at E4.5. Generally, bilateral gonads were not macroscopically evident until E5.5. As development progressed, the left gonads of the female chicken embryos were markedly larger than the right gonads at E7. Subsequently, the dimensions of the left gonads were almost 2.5 times larger than those of the right gonad at E10 (Supplementary Figures S1A–D).

### Transcriptional Profiling

**FIGURE 2:**
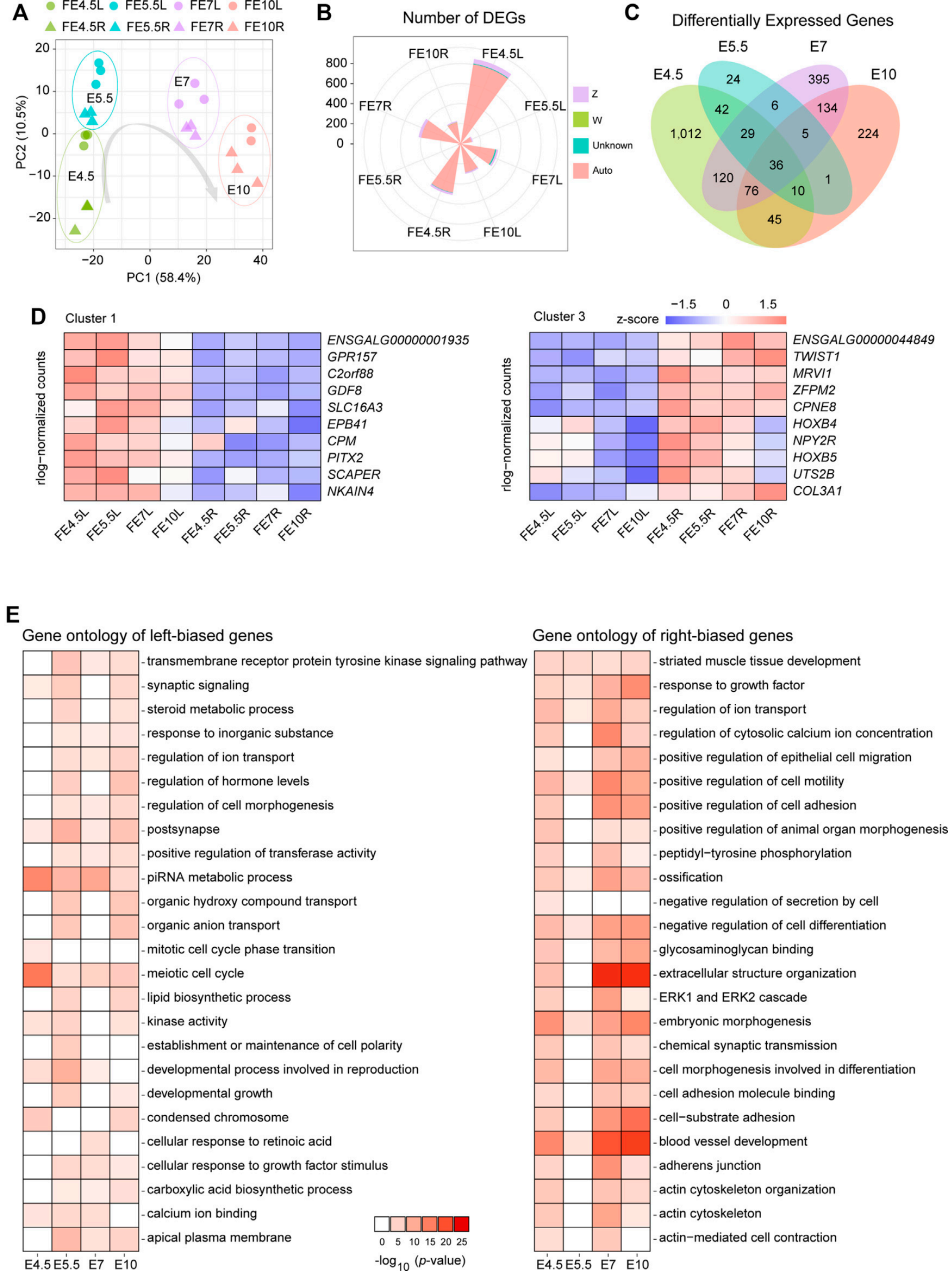
Analysis of RNA-seq data from the left and right gonads. **(A)** PCA plot of RNA-seq data of left and right gonads at different developmental stages; **(B)** The number of DEGs and chromosomal allocation in the left and right gonads; **(C)** Venn diagrams show the shared and unique DEGs obtained from each pairwise comparison between the left and right gonads, including four groups: left gonads and right gonads of female embryonic day 4.5(FE4.5L *vs* FE4.5R), FE5.5L *vs*. FE5.5R, FE7L *vs*. FE7R, and FE10L *vs*. FE10R); **(D)** The gene expression patterns of cluster 1 (left) and cluster 3 (right); **(E)** Top 25 significantly enriched terms of genes among the LBGs (left) and RBGs (right). The depth of the colors represents the size of the *p*-value.

After harvest, gonads were first processed for RNA-seq. The mapping statistics are displayed in Supplementary Table S4. Three replicates of RNA-seq analysis showed high correlation using the Pearson correlation coefficient (Supplementary Figure S2A). The principal component analysis (PCA) plot based on the gene expression profiles exhibited a clear separation between the left and right gonads in each stage (Figure 2A). To identify the differentially expressed genes (DEGs), we conducted a differential expression analysis between left and right gonads in each stage. A total of 855, 123, 378 and 301 left-biased DEGs (LBGs) and 515, 30, 423 and 230 right-biased DEGs (RBGs) were identified at E4.5, E5.5, E7 and E10, respectively (Supplementary Figure S2B; Supplementary Tables S5–S8).

The majority of the DEGs clearly resided on the autosome, and approximately 5% of DEGs were located on the Z chromosome (Figure 2B, Supplementary Figure S2C). The distribution of the DEGs between the bilateral gonads was revealed using a Venn diagram (Figure 2C, Supplementary Figures S1E,F), and 36 common DEGs were identified in the four stages and shown to be located on the autosome (Supplementary Table S9); these common DEGs included 8 TFs (*CALR3*, *CPEB1*, *DMRTB1*, *HOXB4*, *HOXB5*, *PITX2*, *TWIST1* and *ZFPM2*). We identified three clusters of these 36 common DEGs based on a correlation analysis of gene expression (Figure 2D, Supplementary Figure S2D). The heatmap shows the gene expression pattern in the bilateral gonads across the four developmental stages. Clusters 1 and 2 comprise LBGs whose expression levels decreased or increased during the development of the left gonads, and cluster 3 mainly comprises RBGs. The changes in the gene expression *CPM* and *SCAPER* represent characteristic changes in cluster 1, while the dynamic changes in cluster 2 and cluster 3 are represented by changes in *PIWIL1* and *CPNE8* expression, respectively (Supplementary Figure S2E).

To better understand the functional consequences of all the DEGs, enrichment analysis of the LBGs and RBGs identified in all the stages was performed using Metascape software. A total of 25 significantly enriched gene ontology (GO) terms of these DEGs in the bilateral gonads were identified. The significant terms of the LBGs were related to meiotic cell cycle, piRNA metabolic process, regulation of cell morphogenesis, establishment or maintenance of cell polarity, and cellular response to retinoic acid, and these results indicated the role of these genes in sustaining growth and development in left gonads (Figure 2E). Moreover, the terms related to the RBGs were mainly enriched in extracellular structure organization, actin cytoskeleton, cell morphogenesis involved in differentiation and negative regulation of cell differentiation (Figure 2E).

### Chromatin Accessibility Analysis

**FIGURE 3:**
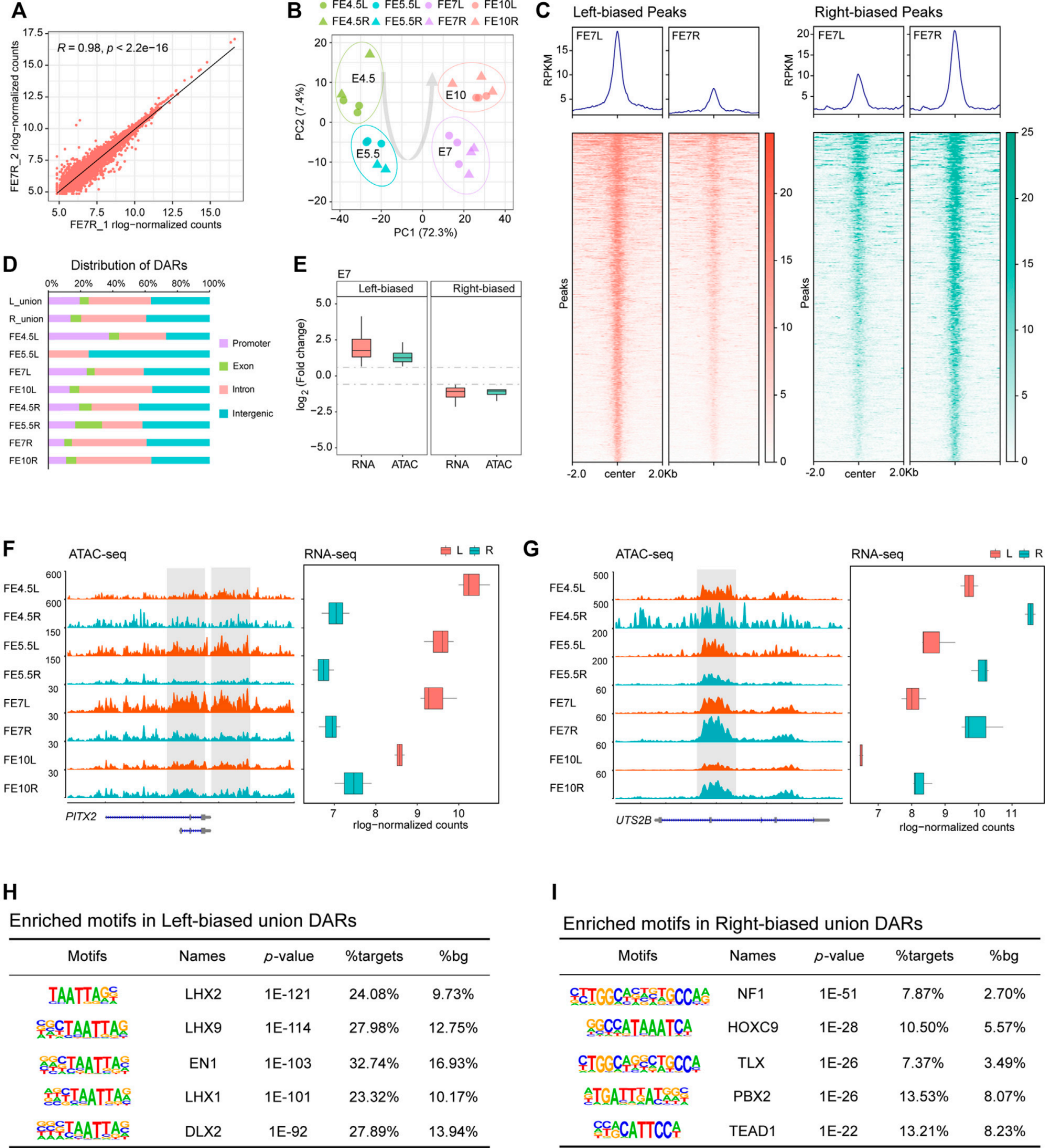
Differential chromatin accessibility between the left and right gonads. **(A)** Scatter plots of sample correlation show high similarities among right gonad duplicates at E7; **(B)** PCA plot of ATAC-seq data of left and right gonads at different developmental stages; **(C)** Bandplots (top) and heatmaps (bottom) showing the quantification of ATAC-seq data of left-biased and right-biased signals in gonads at E7; **(D)** The distribution of DARs in the genome; **(E)** Transcriptional changes in DEGs associated with DARs between the left and right gonads; **(F,G)** Example of left-biased DARs and their associated DEGs (PITX2), as well as right-biased DARs and their associated DEGs (UTS2B). ATAC-seq tracks are shown in the RPKM scale. The *y* axis of the RNA-seq boxplot shows the embryonic day, and the *x* axis shows the mean rlog-normalized counts; **(H)** Top enriched motifs in left union peaks; **(I)** Top enriched motifs in right union peaks.

To understand how genomic regulatory elements control key genes that drive asymmetric gonadal development, we profiled the global chromatin accessibility patterns in both the left and right gonads during each stage using ATAC-seq. Our ATAC-seq data showed high quality in terms of both mapping rate and peak calling (Supplementary Table S10), and the three replicates examined from each stage exhibited great correlation (Figure 3A, Supplementary Figure S3A), indicating strong reproducibility. The PCA plot based on chromatin accessibility showed a clear separation between the left and right gonads in E5.5 and E7 (Figure 3B).

Subsequently, we assessed the differentially accessible regions (DARs), indicated by ATAC-seq signals, between the bilateral gonads in each stage. We identified a total of 3,228 and 3,476 significant DARs in left and right gonadal development, respectively, which are shown by volcano plots (Supplementary Figure S3B). The heatmaps revealed that these significant DARs showed very clear side-specific patterns in each stage (Figure 3C, Supplementary Figures S3C,D). Because few DARs were identified at E5.5 (*n* = 18), only accessibility signals were displayed for the other three stages. Then, the genomic distribution of the DARs in the four developmental stages, the left-biased union DARs and the right-biased union DARs were annotated in their promoter, exon, intron and intergenic regions, and we found that the proportions of the left-biased and right-biased union DARs located in promoters were 19.5 and 13.8%, respectively (Figure 3D). More DARs were located in promoters in the left gonads, suggesting that more genes were activated.

We integrated ATAC-seq and RNA-seq to investigate the relationship between chromatin accessibility and gene expression during asymmetric gonadal development. As expected, we found a strong positive correlation between accessibility signatures and gene expression patterns based on calculated fold changes by assigning open chromatin regions to the nearest DEGs (Figure 3E, Supplementary Figures S3E,F). We found that *PITX2* was highly expressed in the left gonads in the four developmental stages, which was accompanied by striking increases in chromatin accessibility (Figure 3F). Additionally, the gene expression and chromatin accessibility of *UTS2B*, a cell activity-related gene (Prosser et al., 2008), in the right gonads exhibited similar dynamic changes (Figure 3G).

Given that TFs are major factors that drive gene expression by binding to associated regulatory elements, we performed a comparative motif analysis between left-biased and right-biased union DARs using HOMER. We found that the HOXC9 and TEAD gene families were enriched the in right-biased DARs (Figure 3I). Among the left-biased union DARs, the LIM homeodomain (Lhx) family of transcription factors was significantly enriched. Notably, the motif of LHX9, which is related to ovary development in mammals (Birk et al., 2000), was identified as the second most highly enriched motif (Figure 3H).

### The Analysis of LHX9 in Asymmetric Gonadal Development

**FIGURE 4:**
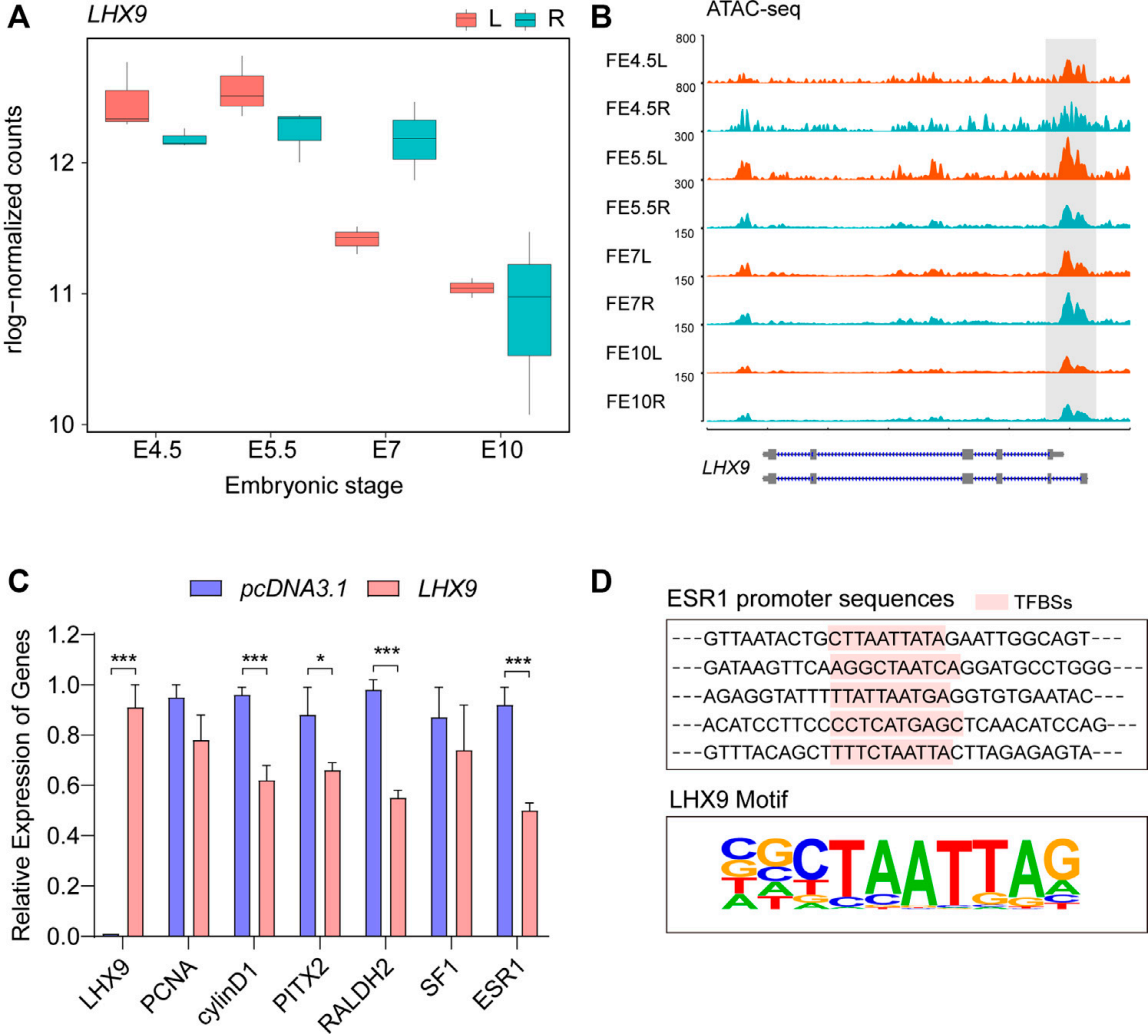
Comprehensive analysis of *LHX9* expression during gonadal development. **(A)** The expression of *LHX9* at each stage. The *x* axis shows the embryonic day, and the *y* axis shows the rlog-normalized counts; **(B)** Chromatin accessibility of the *LHX9* gene at each stage; **(C)** Changes in the relative expression of genes related to asymmetric gonadal development in cells overexpressing *LHX9*; **(D)** The specific TF (LHX9) binding sites (TFBSs) in the promoter regions of *ESR1*.

Considering the important biological functions of LHX9, we first examined the changes in its gene expression in the RNA-seq data to validate our motif screening results. Transcription of the *LHX9* gene remained high but was continuously downregulated in the bilateral differentiated gonads throughout embryonic development (Figure 4A). Notably, compared with the right gonads, the left gonads exhibited significantly lower *LHX9* transcription at E7, suggesting crucial regulatory effects of LHX9 on the developmental trajectory from E5.5 to E7 during gonadogenesis. Moreover, we observed clear differences in chromatin accessibility in the gene body and promoter of *LHX9* (Figure 4B). In light of the differential expression patterns and side-biased motif enrichment, we hypothesized that LHX9 might change its target genes over time by binding to open chromatin regions that change during development.

To validate our motif enrichment results and further explore the role of LHX9 in gonadal development, we used an overexpression vector to regulate the expression of *LHX9* in female left gonadal cells. The qPCR assay was performed to evaluate the effect of LHX9 on gonadal cells at 48 h. We also assessed the expression of *PITX2*, *RALDH2*, *cyclin D1* and *ESR1*, which are involved in asymmetric gonadal development, in cells overexpressing LHX9 and found that their expression levels were significantly downregulated (Figure 4C). Given that *ESR1* is a gene responsible for cortex formation of the left gonad (Guioli et al., 2020), we predicted the potential binding sites of LHX9 in the *ESR1* promoter. Multiple potential LHX9 binding sites were found in the promoter region of *ESR1* (Figure 4D). *ESR1* is a left-biased gene throughout asymmetric gonadal development in female embryos, and chromatin accessibility of *ESR1* promoter increases from E5 to E7 (Supplementary Figure S4). On the basis of these observations, we concluded that LHX9 could affect asymmetric gonadal development by regulating side-biased transcriptional and epigenetic patterns at E7.

## Discussion

Chicken embryos are an excellent model for studying cell biology and embryonic organ development (Davey and Tickle, 2007; Vergara and Canto-Soler, 2012; Seal et al., 2019; Vilches-Moure, 2019). Gonadal development in female chicken embryos is asymmetric, and only the left gonad develops into a functional ovary (Smith and Sinclair, 2004; González-Morán, 2011), and the right gonad remains a steroidogenic organ (Narbaitz and Kolodny, 1964; Samar et al., 1983). This biological phenomenon suggests that regulatory factors related to ovarian development are silent in the right gonads. Therefore, studying the particular mechanisms underlying gonadal development in female chickens is of great significance for studies about sexual development disorders and infertility (Wilhelm and Koopman, 2006; Guercio and Rey, 2014). However, the molecular mechanisms underlying asymmetric gonadal development are still poorly understood. Here, we performed time-course RNA-seq and ATAC-seq experiments in bilateral gonads of female chicken embryos and provided a dynamic profile of transcriptional and chromatin accessibility patterns throughout development. The study elucidated the regulatory network of asymmetric gonadal development and identified several candidate genes and regulatory elements. Significantly, we showed that the LHX9 TF could drive left gonadal development by binding to left-biased DARs and preliminary verified its basic function. The present work provided unique insights into the regulatory mechanisms underlying asymmetric gonadal development.

Due to the limitations of sample availability and sequencing technologies (Wan et al., 2017; Yu et al., 2017; Zou et al., 2020), many previous studies failed to identify the functional genes and elucidate the regulatory mechanisms underlying asymmetric gonadal development. Here, bilateral gonads were harvested from female chicken embryos for sequencing at four developmental stages (E4.5, E5.5, E7 and E10). Each sample library was derived from a single gonad from an independent individual, and this approach is more refined than previous approaches involving the sequencing of pooled samples from multiple individuals. Notably, we obtained sequencing data of bilateral gonads at E4.5; thus, our work is a pioneering study that explored the earliest available stages of gonadal development. Despite the lack of clear differences in morphology between the left and right gonads at E4.5, we observed obvious transcriptional changes, suggesting that the underlying molecular changes that cause developmental discrepancies already occurred. By integrating RNA-seq and ATAC-seq data of female bilateral gonads from different developmental stages, active genes and open chromatin regions were characterized; these findings can contribute to clearly exploring functional genes and detailed regulatory patterns and can provide more accurate guidance for studies of asymmetric gonadal development.

Gonadal morphological differences mainly occur due to increased cell number, proliferation rate and meiosis in the left ovary (Ishimaru et al., 2008; Intarapat and Stern, 2013; de Melo Bernardo et al., 2015). Previous studies proposed that the germ cells were lost by cell death in the right embryonic gonads (Ukeshima, 1996). However, the number of PGCs in the right ovary increases until hatching (González-Morán, 2011), suggesting that any loss in PGC number is compensated by germ cell proliferation. GO analysis showed that the LBGs are mainly enriched in cell proliferation- and cycle-related items, which suggests that LBGs are related to organ development and actively drive gonadal development (Smith et al., 2008a; Rodríguez-León et al., 2008; Thomson and Lin, 2009; Yu et al., 2013; Rengaraj et al., 2014). Previous studies revealed hundreds of side-biased genes in asymmetric gonadal development at the embryonic (E6, E9, and E12) and posthatching stages (D1); these genes include *DAZL*, *GDF8*, *PITX2*, *PIWIL1*, and *TDRD5* (Intarapat and Stern, 2013; Wan et al., 2017; Zou et al., 2020), and these findings are consistent with our work. As the factor that determines left-right bias in vertebrates (Amand et al., 1998; Piedra et al., 1998; Yoshioka et al., 1998; Schweickert et al., 2000; Dagle et al., 2003; Gormley and Nascone-Yoder, 2003; Grimes and Burdine, 2017; Hamada, 2020; Little and Norris, 2021), PITX2 has been proven to play a key role in gonadal lateralization and morphogenesis by regulating *RALDH2*, *SF1* and *cyclin D1* expression, promoting cell proliferation and reorganizing the cytoskeleton. Besides, overexpression of PITX2 can rescue the degeneration of the right ovary during gonadogenesis (Guioli and Lovell-Badge, 2007; Ishimaru et al., 2008; Rodríguez-León et al., 2008). Based on gene expression in the four developmental stages, we identified a cluster of genes among 36 common DEGs whose expression was most highly correlated with PITX2 expression. The heatmap of cluster 1 pattern revealed that the gene expression of left gonad increased during development, which may be indicative of its facilitation actions in left gonadal development. Among these genes, we found that the *SCAPER*, *CPM* and *GDF8* genes were related to cell cycle progression, differentiation, and proliferation (Rehli et al., 1995; McPherron et al., 1997; Tsang et al., 2007), which further confirmed and narrowed the scope of candidate genes. Nevertheless, the exact roles of these candidate DEGs during asymmetric gonadal development require further investigations. In short, we provided a distinct regulatory network of asymmetric gonadal development.

Although some advances have been made in assessing core genes related to asymmetric gonadal development, the upstream regulatory elements remain largely unknown. To fill this knowledge gap, ATAC-seq experiments were used to identify key regulatory drivers responsible for gonadal development. Studying dynamic changes in chromatin accessibility in gene regulatory regions during transcriptional activation provides insight into changes in cell fate. Interestingly, the PC1 in both RNA-seq and ATAC-seq PCA plots separated the samples into undifferentiated and differentiated. Additionally, E5.5 showed fewer DEGs and DARs than the rest of them, which means that the ovarian asymmetry is strongly induced after the onset of sex differentiation (E6). A previous study showed that the formation of the embryonic gonadal cortex was induced by estrogen (Guioli et al., 2020). Indeed, the estrogen synthesis was induced by the aromatase in the female chick embryo (Nakabayashi et al., 1998; Lambeth et al., 2016). Aromatase shows sexual dimorphism and gonads begin morphological differentiation into testes or ovaries at E6 in the chicken embryos (Ayers et al., 2015), which means that the ovary of chicken embryos produced estrogen from the start of female-specific differentiation (Guioli et al., 2020). Thus, the unique side-dependent developmental pattern of female bird gonad was affected by sex determination or differentiation pathways. Integration of RNA-seq and ATAC-seq data can facilitate the characterization of the chromatin accessibility and gene expression patterns that are pivotal for asymmetric gonadal development. In this study, the RNA-seq data showed that more DEGs were identified in the left gonad, which is consistent with the high proportion of left-biased DARs in promoter regions as shown by ATAC-seq. This result indicated that more genes remained active in the left gonad, thereby promoting left gonadal development. In general, gene expression is regulated by TFs that bind to DNA in open chromatin regions (Thurman et al., 2012; MacNeil et al., 2015). Thus, we predicted putative TF binding motifs in DARs. HOXC9, which acts as a regulator of cell proliferation and cycle related gene expression and as a mediator of RA action in neuroblastoma cells (Mao et al., 2011; Stoll et al., 2011; Wang et al., 2013), was the second most highly enriched motif in the right-biased DARs. In addition, the TEAD gene family, which includes core members of the Hippo pathway, functions mainly to inhibit proliferation and to promote apoptosis, thereby limiting the overgrowth of organs (Harvey and Tapon, 2007; Harvey et al., 2013); members of this gene family were also enriched in the right-biased DARs. Notably, the TFs of the LIM homeobox gene family were enriched in the left-biased DARs, which suggested that the LHX family probably binds to open chromatin regions during gonadal development. Several previous works suggested that the LIM homeobox gene family plays key roles in regulating gene expression patterns throughout the body during development in invertebrates and vertebrates (Curtiss and Heilig, 1998).

Differential motif analysis confirmed that LHX9 preferentially bound to open chromatin regions during left gonadal development. Interestingly, LHX9, which is required for gonadogenesis in mice, promotes the proliferation of the early somatic cell population (Birk et al., 2000). However, previous studies did not sufficiently elucidate the function of LHX9 in asymmetric gonadal development (Guioli and Lovell-Badge, 2007; Ishimaru et al., 2008). A recent study showed that LHX9, which is strongly expressed in the cortex overlying chicken gonads, is a downstream target of hedgehog signaling (the upstream pathway most closely involved in triggering and orchestrating gonadogenesis in chickens) (Yoshino et al., 2016); these results indicated that LHX9 is required in the normal development of gonads. The expression of the TF that regulates LHX9 expression remains constant throughout bilateral gonadal development; however, the TF has a distinct preference for binding the genome in the left gonads, suggesting that chromatin accessibility determines local affinity for the LHX9 gene. Research has suggested that prior to the binding of other TFs, pioneer TFs recognize specific sequences in DNA binding sites and directly recruit chromatin-remodeling complexes, actively increase or decrease local chromatin accessibility, and then precisely control gene expression (Zaret and Carroll, 2011). The switch in LHX9 binding sites may explain how LHX9 performs different functions in bilateral gonads.

Our *in vitro* experiments revealed that genes related to asymmetric gonadal development were altered with *LHX9* overexpression, which means that LHX9 may target these genes to regulate gonadal development at E7. Previous work showed that the ovarian L-R asymmetry is strongly induced by estrogen, which acts as the major promoting signal for cortex development. ESR1 (ERα), depending on its activity within the left epithelial cells, is the main transducer of estrogen signal in cortex formation of the left gonad (Guioli et al., 2020). Interestingly, *ESR1* is a left-biased gene throughout asymmetric gonadal development in female embryos, and chromatin accessibility of *ESR1* promoter increases from E5 to E7. As development progressed, the left gonads of the female chicken embryos were markedly larger than the right gonads until E7, which accompanied by the left gonads exhibited significantly lower LHX9 transcription. In addition, ERα and LHX9 co-localize only in cortical cells of the left gonad at E7 (Guioli and Lovell-Badge, 2007). Further, we predicted the potential binding sites of LHX9 in the *ESR1* promoter using Homer. As expected, we found multiple LHX9 potential binding sites in the promoter regions of *ESR1*. *ESR1* was downregulated by LHX9 in female gonad cells at E7, which suggested that LHX9 could act upstream of ESR1. Thus, decreased transcription of LHX9 on the left side, especially at E7, should be an important factor that drives asymmetric gonadal development *via* regulating ESR1 activity. It is necessary for future research to confirm the regulation and function of LHX9 *in vivo* in gonadal epithelium formation through overexpressing and knocking down/out experiments. Bulk RNA-seq mainly reflects the averaged gene expression across thousands of cells. Gonad RNA-seq could not reveal the subtle spatio-temporal transcriptional pattern of LHX9 and ESR1 in gonadal epithelial cells, since the two candidates might express in only a certain cell type. Single-cell RNA-sequencing technologies can study cell heterogeneity and analyze gene expression at individual cells, which may contribute to a better understanding of the role of LHX9 in chicken embryonic gonads. Further study at the single-cell level would be more informative to explore the distribution of LHX9 in different gonadal lineages. Integrating matched RNA-seq, ATAC-seq and single cell RNA-seq will be helpful to elucidate molecular basis underlying asymmetric gonadal development. Although the function of LHX9 in asymmetric gonadal development is preliminarily understood, the affinity of LHX9 for local chromatin is still not very clear. Therefore, follow-up work should focus on exploring the regulation of pioneer TFs that in turn regulate chromatin.

Previous studies have demonstrated the effect of transcription factors on asymmetric development. Here, we first revealed the contribution of dynamic changes in chromatin accessibility to these processes. In summary, we established a high-resolution profile of transcriptional and chromatin accessibility patterns during chicken gonadal development, revealed a functional relationship between chromatin accessibility and candidate gene expression, and identified a promising TF, LHX9*,* that may regulate the left gonad to develop into a functional ovary. Together, our findings will enable a better understanding of ovarian development after sex determination and the mechanisms underlying asymmetric patterns of development in vertebrates. Additionally, our findings can also serve as a potential guide for establishing biological models to probe the causes of ovary-related diseases in humans.

## Data Availability

The original contributions presented in the study are publicly available. This data can be found here: National Center for Biotechnology Information (NCBI) BioProject database under accession number PRJNA756705.
