# Supplementary material for: Time-Course Transcriptional and Chromatin Accessibility Profiling Reveals Genes Associated With Asymmetrical Gonadal Development in Chicken Embryos
Source: Front Cell Dev Biol. 2022 Mar 8;10:832132. doi: 10.3389/fcell.2022.832132 (PMC8957256; doi:10.3389/fcell.2022.832132)
Supplement: Supplementary file 1 [file Table2.DOCX]

Supplementary Material

# Supplementary Figures


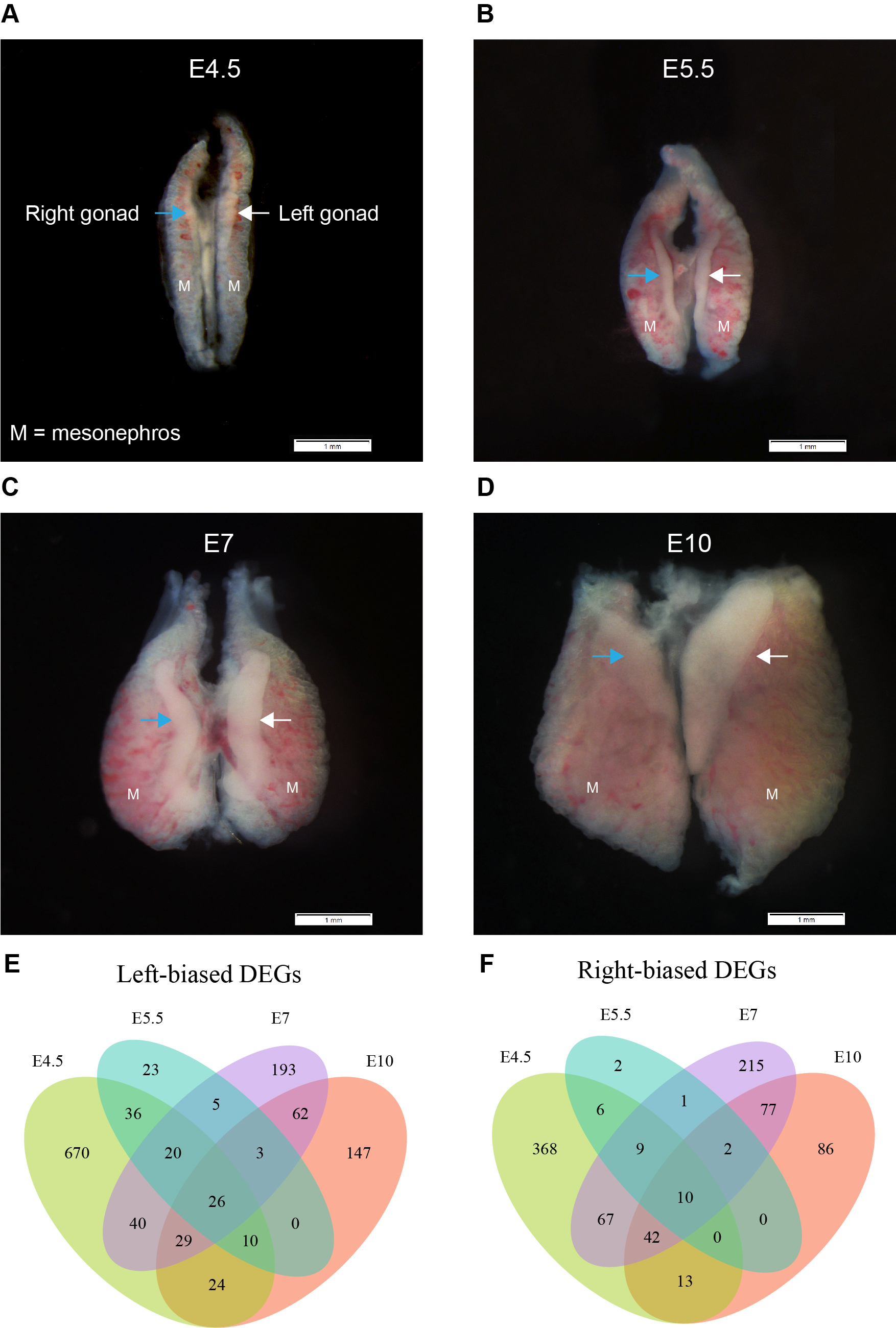


**Supplementary Figure S1.** Gross morphology of developing female chicken gonads. **(A-D)** Ventral view of gonads and mesonephros from day 4.5 to day 10 of development. Left and right gonads are indicated by white and blue arrows, respectively. The left and right gonads were similar in size in the early period (E4.5 and E5.5). In the later stages of development (E7 and E10), the left gonads were larger than the right gonads in females; **(E-F)** Venn diagrams show the shared and unique left-biased and right-biased DEGs obtained from each pairwise comparison between the left and right gonads, including four groups: left gonads and right gonads of female embryonic day 4.5(FE4.5L vs FE4.5R), FE5.5L vs. FE5.5R, FE7L vs. FE7R, and FE10L vs. FE10R).

**
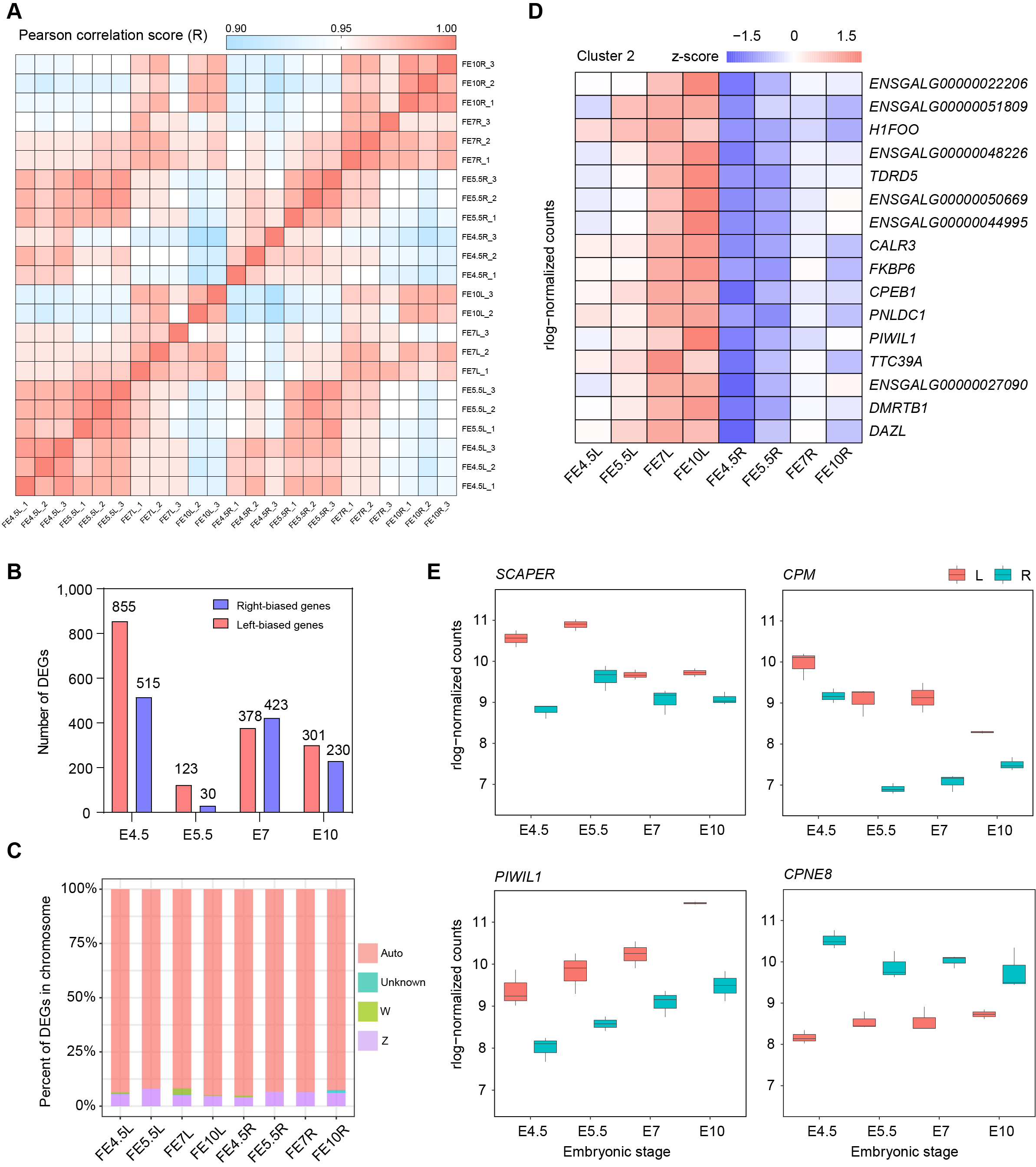
**

**Supplementary Figure S2.** Analysis of DEGs between the left and right gonads. **(A)** The correlation matrix of RNA-seq samples; **(B)** The number of DEGs between bilateral gonads in each stage; **(C)** The percent of DEGs in each chromosomal allocation between the left and right gonads; **(D)** The gene expression pattern of cluster 2; **(E)** The gene expression pattern of typical DEGs.

**
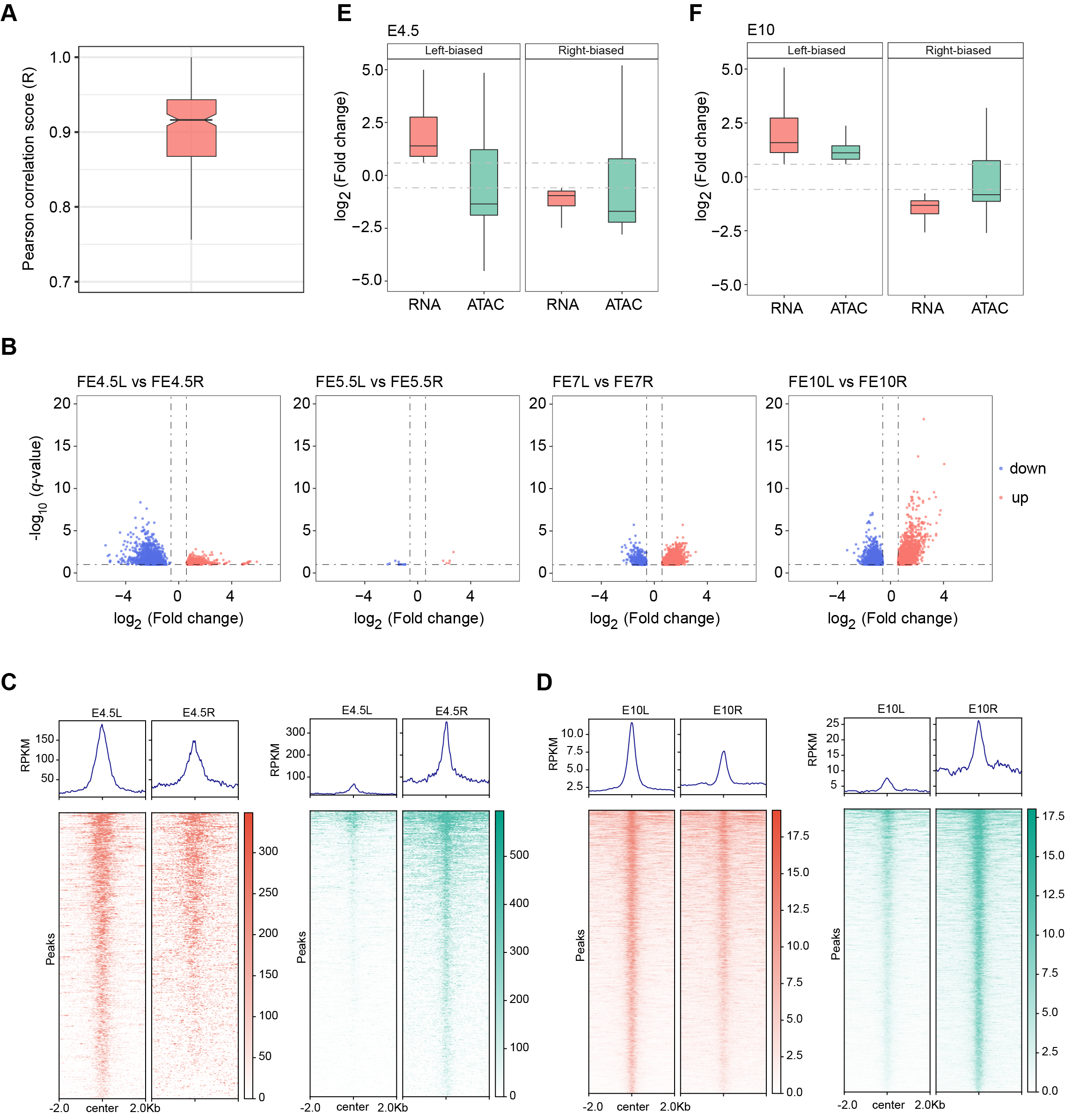
**

**Supplementary Figure S3.** The statistical analysis of ATAC-seq data. **(A)** The correlation score of the ATAC-seq samples; **(B)** Volcano plots of ATAC-seq comparisons between the left and right gonads at four developmental stages. The x axis shows the log2-fold change in the reads detected at open chromatin regions, and the y axis shows − log10 (*q*-value); **(C-D)** Bandplots (top) and heatmaps (bottom) showing the quantification of the ATAC-seq data of left-biased and right-biased signals in the gonads at E4.5 (C) and E10 (D); **(E-F)** Transcriptional changes in DEGs associated with DARs between the left and right gonads at E4.5 (E) and E10 (F).


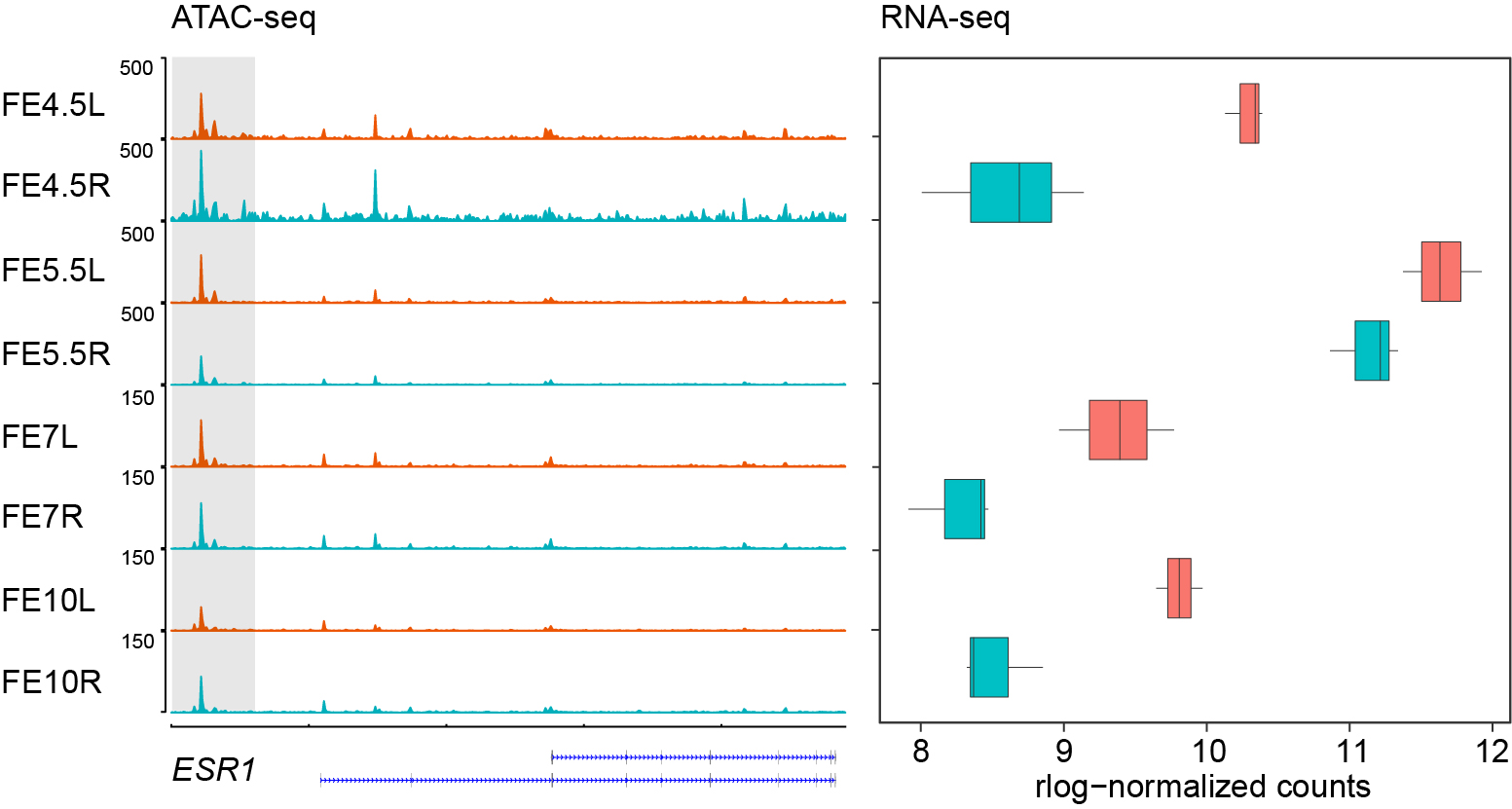


**Supplementary Figure S4.** Chromatin accessibility (left) and gene expression (right) of ESR1 at each stage. ATAC-seq tracks are shown in the RPKM scale. The y axis of the RNA-seq boxplot shows the embryonic day, and the x axis shows the mean rlog-normalized counts;
